# Extending the Cultivation Area of Pecan (*Carya illinoinensis*) Toward the South in Southeastern Subtropical China May Cause Increased Cold Damage

**DOI:** 10.3389/fpls.2021.768963

**Published:** 2021-11-30

**Authors:** Jinbin Zheng, Heikki Hänninen, Jianhong Lin, Sitian Shen, Rui Zhang

**Affiliations:** ^1^State Key Laboratory of Subtropical Silviculture, Zhejiang A&F University, Hangzhou, China; ^2^SFGA Research Center for Torreya Grandis, Zhejiang A&F University, Hangzhou, China

**Keywords:** chilling requirement, climatic warming, experimental ecophysiology, leaf-out, pecan, process-based tree phenology models, scenario simulations, subtropical trees

## Abstract

Pecan (*Carya illinoinensis*) is an important nut tree species in its native areas in temperate and subtropical North America, and as an introduced crop in subtropical southeastern China as well. We used process-based modeling to assess the effects of climatic warming in southeastern China on the leaf-out phenology of pecan seedlings and the subsequent risk of “false springs,” i.e., damage caused by low temperatures occurring as a result of prematurely leafing out. In order to maximize the biological realism of the model used in scenario simulations, we developed the model on the basis of experiments explicitly designed for determining the responses modeled. The model showed reasonable internal accuracy when calibrated against leaf-out observations in a whole-tree chamber (WTC) experiment with nine different natural-like fluctuating temperature treatments. The model was used to project the timing of leaf-out in the period 2022–2099 under the warming scenarios RCP4.5 and RCP8.5 in southeastern China. Two locations in the main pecan cultivation area in the northern subtropical zone and one location south of the main cultivation area were addressed. Generally, an advancing trend of leaf-out was projected for all the three locations under both warming scenarios, but in the southern location, a delay was projected under RCP8.5 in many years during the first decades of the 21st century. In the two northern locations, cold damage caused by false springs was projected to occur once in 15–26 years at most, suggesting that pecan cultivation can be continued relatively safely in these two locations. Paradoxically, more frequent cold damage was projected for the southern location than for the two northern locations. The results for the southern location also differed from those for the northern locations in that more frequent cold damage was projected under the RCP4.5 warming scenario (once in 6 years) than under the RCP8.5 scenario (once in 11 years) in the southern location. Due to the uncertainties of the model applied, our conclusions need to be re-examined in an additional experimental study and further model development based on it; but on the basis of our present results, we do not recommend starting large-scale pecan cultivation in locations south of the present main pecan cultivation area in southeastern subtropical China.

## Introduction

The timing of phenological events of trees in spring, such as leafing out of vegetative buds, is crucial for the survival and growth of trees (Fuchigami et al., 1982; Hänninen, 2016; Perez-de-Lis et al., 2016). At the ecosystem level, the spring phenology of trees regulates several key processes, such as the cycling of carbon and nutrients and productivity of forests and plantations (Piao et al., 2009; Keenan et al., 2014; Zhou et al., 2020). In the temperate zone, analyses of long-term phenological records have shown a general advancement of spring phenology over the past few decades caused by global warming (Richardson et al., 2013; Vitasse et al., 2018). However, several cases have also been found where the advancement has been leveling off (Fu et al., 2015).

Air temperature is a major environmental factor regulating the spring phenology of trees in boreal and temperate zones. It has a dual role (Sarvas, 1972, 1974; Polgar and Primack, 2011). First, after growth cessation and bud set in the autumn, long-term exposure to chilling temperatures is required for a rest break, synonymous to endodormancy release (Lang et al., 1987), which means the removal of growth-arresting physiological conditions within the buds (Fuchigami et al., 1982; Cooke et al., 2012; Hänninen et al., 2019). Temperatures in the range of 0–10°C have traditionally been regarded as the most effective in causing rest breaks (Sarvas, 1974; Hänninen, 2016). Second, after the chilling requirement of rest completion is met, the period of quiescence, synonymous with ecodormancy (Lang et al., 1987), is attained. In quiescence, bud burst is prevented only by environmental factors, typically low air temperatures, so that it takes prolonged exposure to relatively high forcing temperatures to cause the occurrence of visible phenological events, such as leafing out and flowering (Sarvas, 1972, 1974). Besides air temperature, photoperiod has long been considered to regulate the spring phenology of trees, at least in some species (Wareing, 1953; Caffarra and Donnelly, 2011; Vitasse and Basler, 2013); more recently, further evidence for the role of photoperiod has been published (Basler and Körner, 2012; Fu et al., 2019; Meng et al., 2021).

The past few decades have seen a growing trend of constructing process-based models of the spring phenology of boreal and temperate trees. These models address the developmental phenomena taking place during periods of rest and quiescence with explicit state variables (Hänninen, 2016; Chuine and Régnière, 2017). Recently, it has been shown experimentally that subtropical trees also show a rest period and chilling requirement (Du et al., 2019; Song et al., 2020; Jewaria et al., 2021; Pan et al., 2021; Zhang et al., 2021a, b). The first process-based models of spring phenology have been published for subtropical trees (Chen et al., 2017; Zhang et al., 2021c).

Since the 1980s, process-based models of the spring phenology of trees have been applied to project the effects of climatic warming on boreal and temperate trees (Cannell, 1985; Hänninen, 1991; Vitasse et al., 2011; Chuine et al., 2016; Ford et al., 2016; Wang et al., 2020). Climatic warming may have two different adverse effects on the spring phenology of trees. First, especially under warm climates, reduced chilling under climatic warming may delay the spring leaf-out and flowering (Murray et al., 1989), and the warming may even cause abnormal development of vegetative and flower buds in the most severe cases, thus reducing the growth and production of fruits (Erez, 2000; Man et al., 2017). Second, warming may advance the spring leaf-out and flowering to the extent that the risk of damage caused by low spring temperatures increases (Cannell, 1985; Hänninen, 1991). This phenomenon, more recently called “false spring” (Marino et al., 2011; Chamberlain et al., 2019), may cause considerable damage even under the present climate (Gu et al., 2008; Kaur et al., 2020). However, if the advancement of spring phenology is not accompanied by the increased incidence of cold damage, then climatic warming may increase the productivity of trees and forests by prolonging the growing season (Kramer and Hänninen, 2009). In all, trustworthy projection of the effects of warming on the spring phenology of trees calls for biologically realistic process-based models.

Pecan (*Carya illinoinensis*) was introduced to China about a 100 years ago from the United States, where it has a broad geographical range in both the temperate and subtropical zones. Now it is a major horticultural crop in subtropical southeastern China (Zhang et al., 2015). Sparks (1993) showed that pecan evinces a rest period and a chilling requirement of a rest break. Here, we developed a process-based model for spring leaf-out in seedlings of pecan grown in southeastern subtropical China. We took an experimental approach, where model development was based on growth chamber experiments explicitly designed to examine the processes addressed by the models. The model developed was applied to project the timing of pecan seedling leaf-out and the subsequent risk of cold damage under two climatic scenarios. In the simulations, we examined which one of the three hypotheses was projected to be realized in pecan seedlings grown in three locations in southeastern China: (1) leaf-out is delayed as a result of reduced chilling (the delay hypothesis), (2) leaf-out is advanced to the extent of causing the increased occurrence of cold damage (the false spring hypothesis), and (3) leaf-out is advanced without increased incidence of cold damage (the prolonged growing season hypothesis).

## Materials and Methods

### Structure of the Overall Model

The process-based spring phenology model for pecan leaf-out was developed by applying a modular model structure consisting of three sub-models (Supplementary Figure 1; Hänninen, 1990, 2016; Hänninen and Kramer, 2007; for recent applications of the approach, refer to Lundell et al., 2020; Zhang et al., 2021c). Sub-models I and II correspond to the various chilling and forcing accumulation models (Kramer, 1994; Chuine et al., 1998), respectively, but in order to emphasize the physiological phenomena modeled, we use the biological names of the rate and state variables here (Hänninen, 1995, 2016; Hänninen et al., 2019; Supplementary Figure 1).

Sub-model I addresses the effects of the chilling temperatures on the rate of a rest break, R_t_(t) (Supplementary Figure 1). The state of rest break, S_r_(t), is obtained by integrating R_r_(t) with respect to time from the onset of rest, t_0_, to time instant t. R_t_(t) is scaled on a percentage scale, so that rest completion is predicted to take place when S_t_(t) = 100%. Sub-model II addresses the ontogenetic development, i.e., the microscopic anatomical changes within the bud that lead to the visible phenological event, such as leafing out. In particular, Sub-model II addresses the effects of high forcing temperatures on the potential rate of ontogenetic development, R_o,pot_(t) (Supplementary Figure 1). The potential rate indicates the rate after rest completion when the rate is no longer restricted by the rest status of the bud.

The restrictions caused by the rest status are addressed by sub-model III, where the value of a theoretical variable, ontogenetic competence, C_o_(t), is calculated as a function of the state of rest break, S_r_(t). C_o_ is a [0,1]-multiplier mediating the effects of rest status to the (realized) rate of ontogenetic development, R_o_(t) (Supplementary Figure 1). With C_o_(t) = 0, no ontogenetic development takes place regardless of the ambient temperature. At rest completion with R_t_(t) = 100%, the value ontogenetic competence is C_o_(t) = 1 by definition. Otherwise, the relationship between C_o_(t) and S_r_(t) can take different forms, making it possible to address contrasting models, such as the parallel model and the sequential model, within the same framework (Hänninen, 1990, 2016). Finally, the state of ontogenetic development, S_o_(t), is obtained by integrating R_r_(t) with respect to time from the onset of rest, t_0_, to time instant t. Leaf-out is predicted to occur when S_o_(t) = 100%.

### Experimental Designs and Conditions

#### Autumn Experiment

In the autumn experiment, the effects of chilling on rest break and, further, on ontogenetic competence were examined (sub-models I and III, Supplementary Figures 1, 2). The experimental seedlings were first exposed to chilling for periods of varying duration, either under natural conditions outdoors or under controlled conditions in growth chambers, and then transferred to high-temperature forcing conditions in growth chambers. In the latter, a regrowth test was carried out by observing the occurrence and timing of leaf-out (for details, refer to Supplementary Material). The controlled chilling treatment was carried out for the purpose of formulating sub-models I and III (Supplementary Figure 1). The natural chilling treatment was carried out to determine the timing of rest completion under natural conditions. This information was used to determine the timing of the spring experiment, whose results were used in formulating sub-model II.

The controlled chilling took place in computer-controlled growth chambers (E-Lotus Technology Co., Beijing, China): air temperature +6°C, constant day length 10 h 30 min, with the light period from 6:30 am to 5 pm, and a relatively low photosynthetic photon flux density (PPFD) (150 μmol m^–2^ s^–1^). The conditions in the controlled chilling approximated those typical for the winter months in the area. Two chilling chambers (2.55 m × 2 m × 2.5 m) were used in the experiment. Both chambers had eight shelves (0.6 m × 1.1 m) with LED lights located above the shelves. The forcing took place in computer-controlled growth chambers (E-Lotus Technology Co., Beijing, China): air temperature +20°C, constant day length 12 h, with the light period from 6.30 am to 6.30 pm, and a relatively high PPFD (400 μmol m^–2^ s^–1^). The conditions in the forcing chambers approximated those prevailing in the area at the spring equinox, some weeks before the leaf-out of most tree species is observed under natural conditions. Two forcing chambers (4.92 m × 2 m × 2.5 m) were also used in the study. Both chambers had four shelves (0.6 m × 2.3 m) with LED lights located above the shelves. In both the chilling and the forcing chambers, the relative humidity was 70–80%, concentration was CO_2_ 300–400 ppm, and air circulation was carried out four times a day for 20 min each time. The seedlings were watered every 3 days in the chilling chambers and every 2 days in the forcing chambers.

At the beginning of the experiment, on November 21, 2017, 10 seedlings were sampled from the outdoor seedling collection for the forcing conditions (0 weeks chilling). Out of the remaining 120 seedlings, one-half were sampled for controlled chilling in the growth chambers, while the other half remained under natural chilling conditions outdoors. After that, 10 seedlings from both chilling conditions were sampled at 2-week intervals for the forcing conditions. The last transfer was carried out on February 12, 2018, so the duration of chilling varied from 0 to 84 days. Under the forcing conditions, leaf-out was inspected at intervals of 2 or 3 days (for details, refer to Supplementary Material). The leaf-out observations under the forcing conditions were stopped on April 23, 2018.

In the analysis of the results, the generic term “bud burst” was used for leaf-out (Zhang et al., 2021a). This was done in order to report the results in terms of standard indices bud burst percentage, BB%, and days to bud burst, DBB. Accordingly, for each treatment group, defined by the duration (0–84 days) and type (natural, controlled) of chilling, BB% was calculated as the percentage of seedlings showing leaf-out. Additionally, for each seedling showing leaf-out, DBB was determined as the time required under the forcing conditions for leaf-out. In the analysis of the results, both BB% and DBB were plotted against the duration of chilling.

#### Spring Experiment

In the spring experiment, the effects of relatively high air temperatures (“forcing”) on the timing of leaf-out were examined (sub-model II; Supplementary Figures 1, 2). After overwintering under natural conditions, 10 seedlings were sampled from the natural outdoor conditions for each of the three growth chambers (E-Lotus Technology Co., Beijing, China) with constant air temperatures of +10, +17, and +24°C. The environmental conditions other than air temperature were the same as in the autumn experiment: 12-h day length, PPFD 400 μmol m^–2^ s^–1^, relative humidity 70–80%, and concentration of CO_2_ 300–400 ppm. Leaf-out was observed in each chamber every 3 days. The days to bud burst (leaf-out), DBB, was recorded for each experimental seedling, and in the analysis of the results, the observations were plotted against the experimental temperature. The data were used for formulating sub-model II (air temperature response of the potential rate of ontogenetic development). For such a purpose, the experiment should optimally be started after rest is completed, but there should be no occurrence of considerable ontogenetic development caused by high temperatures under natural conditions. If such ontogenetic development had already occurred under the natural conditions before the start of the experiment, that would decrease the value of DBB and, thereby produce erroneous data for the modeling. This optimal timing was not known *a priori*, which is why the experiment was repeated at three different times by transferring a sample of the seedlings from the natural outdoor conditions to the three growth chambers each time. The transfer dates were February 13, 2017, February 27, 2017, and March 13, 2018. For independent model testing, leaf-out was additionally observed in a control group of 10 seedlings that remained under natural conditions throughout the experiment.

#### Whole-Tree Chamber Experiment

In the whole-tree (WTC) experiment, the effects of autumn, winter, and spring temperatures on the timing of leaf-out were examined under conditions similar to those occurring naturally. All the treatments were based on the natural ambient temperature, and natural photoperiod was applied (for details, refer to below). The results of the WTC experiment were used for estimating the parameters t_0_ and T_upp_ (refer to the section “Constructing the sub-models on the basis of experimental data” below) and for testing the accuracy of the overall model (internal validation) (Supplementary Figure 2).

Three temperature levels were included: ambient, ambient +2°C, and ambient +4°C. The experiment was started on November 10, 2019, and for the definition of our treatments, we denoted “winter” as the period of November 10, 2019, to February 10, 2020. The time after that, until the observed leaf-out, was denoted as “spring.” A factorial 3 × 3 design was adopted for the experiment, such that each of the three levels of winter temperature was combined with the corresponding three levels of spring temperature, thus creating a total of nine treatments (Supplementary Figure 2). In each treatment, there were eight replicated seedlings.

The experiment was implemented with three transparent whole-tree chambers (WTCs, E-Lotus Technology Co., Beijing, China). The chambers have a smart temperature control system to keep the temperature at the intended level. In one chamber, the air temperature was kept at the ambient level. In the other two WTCs, the air temperature was elevated by 2 and 4°C, respectively, above the ambient temperature. The experiment was implemented by (1) placing the seedlings of each treatment group in the chamber representing the intended winter temperature at the beginning of the experiment, and (2) transferring the seedlings on February 11, 2020, to the intended spring temperature (Supplementary Figure 2). In order to avoid any systematic errors caused by possible temperature gradients within the chambers, the locations of the seedlings in the chambers were rotated constantly during the experiment.

### Statistical Analysis of the Experimental Results

In the autumn experiment, the differences in BB% between the treatments were analyzed by means of logistic regression with a binary response (bud burst: no/yes) using the type (natural vs. controlled in +6°C) and duration of chilling as the explaining factors. The differences in DBB between the treatments were analyzed by means of a two-way ANOVA using the same explaining factors as with BB%. A two-way ANOVA was also applied to analyze the differences in DBB between the treatments in the spring experiment. The explaining factors were the time of transfer from natural conditions to forcing conditions and the temperature under the forcing conditions.

### Constructing the Sub-Models on the Basis of Experimental Data

#### A Framework for Formulating the Models on the Basis of Experimental Data

The three sub-models were constructed on the basis of experimental data, following the methodological framework introduced by Zhang et al. (2021c), with few modifications. The air temperature responses described by the three sub-models were inferred on the basis of their implications for the responses to the occurrence and timing of the readily observable leaf-out under the experimental conditions. Because of these peculiarities of the phenomena modeled, the use of experimental data involved two steps: first, the empirical data points were calculated by analyzing the experimental leaf-out data. Second, the data points were plotted against the respective explanatory variable, and a response curve was fitted to the data.

#### Sub-Model I

For any constant temperature T′, the empirical value of the rate of rest break was calculated as (Sarvas, 1974; Zhang et al., 2021c)


(1) $$R_{r}\left(T^{\prime }\right)=100\frac{1}{\Delta t\left(T^{\prime }\right)}$$

where, Δt = the duration of chilling required for rest completion in the autumn experiment. As a result of using the multiplier 100, the scale for the rest break is the percentage scale [0,100%], so the value of R_r_ calculated with Equation (1) indicates the percentage, out of the total number of physiological reactions required for rest completion, of such reactions taking place in 1 h. The value of Δt was determined in terms of days at first but then converted to hours by multiplying the original value by 24 because the model was applied with a time step of 1 h.

The requirement set for rest completion was that in the regrowth test under forcing conditions the bud burst (leaf-out) percentage, BB%, is at or near 100% and that the curve representing days to bud burst (leaf-out), DBB, as a function of the duration of chilling levels off, indicating that the rate of ontogenetic development cannot be substantially increased by further chilling. Following Zhang et al. (2021c), we determined Δt for the controlled chilling treatment in +6°C by requiring that the BB% is at least 80% and by determining the leveling off of the DBB curve as follows: first, an exponential curve was fitted to the scatter plot representing the DBB of each of the seedlings as a function of the duration of chilling:


(2) $$\text{y}\left(\text{x}\right)=f_{1}\left(\text{x}\right)=y_{0}+a_{1}{\text{e}}^{-b_{1}\text{x}}$$

where, y = the modeled value of days to bud burst; x = the duration of chilling (days); and y_o_, a_1_, and b_1_ are parameters to be estimated. Then, in order to study the slope of the DBB curve, its first derivative with respect to the duration of chilling, x, was calculated as follows:


(3) $$\frac{dy}{dx}=-a_{1}b_{1}e^{-b_{1}x}$$

**FIGURE 1:**
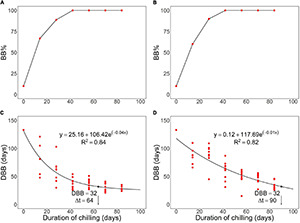
Effects of chilling on a rest break in first-year pecan seedlings in the autumn experiment, where both controlled chilling in constant +6°C **(A,C)** and chilling outdoors under natural conditions **(B,D)** were used. The rest break caused by prolonged chilling is manifested after the transfer to the growth-promoting forcing conditions as an increase in panels **(A,B)** bud burst percentage, BB% and a decrease in panels **(C,D)** days to bud burst (DBB). The arrow and the index Δt indicate the estimated number of days required for meeting the chilling requirement of rest completion under the chilling conditions. The black dot indicates the corresponding value of the DBB at the time of rest completion. Note that the phenological event observed was leaf-out but that the concept “bud burst” is used here as a generic one facilitating the use of the standard indices BB% and DBB.


Following Zhang et al. (2021c), we determined Δt as equal to the duration of chilling x, where the value of the first derivative is equal to −0.3 (refer to Figure 1C in the section “Results”):

(4) $$\frac{dy}{dx}=-a_{1}b_{1}e^{-b_{1}x}=-0.3$$

implying


(5) $$\Delta \text{t}=\text{x}=-\frac{1}{b_{1}}\ln\frac{0.3}{a_{1}b_{1}}$$

Finally, the empirical value of the rate of rest break in +6°C was calculated by plugging the value of Δt obtained with Equation (5) for that treatment into Equation (1).

Due to the limited growth chamber facilities, we were not able to include other controlled constant chilling temperatures beside the +6°C used in the study. In the absence of experimental data for other temperatures, we determined sub-model I as follows (Lundell et al., 2020): (1) a plateau temperature response in the temperature range from the lower threshold temperature of −3.4°C (Sarvas, 1974) to the upper threshold temperature, T_upp_, and (2) no rest break taking place below the lower or above the upper threshold (refer to Figure 3A in the section “Results”):


$$R_{r}\left(T\right)=0,whenT<-3.4^{{}^{\circ}}\text{C}$$

$$R_{r}\left(T\right)=R_{r,obs},when-3.4^{{}^{\circ}}\text{C}\leq T<T_{upp}$$

(6) $$R_{r}\left(T\right)=0,whenT\geq T_{upp}$$

where, R_r_(T) is the modeled rate of a rest break, T(t) is the air temperature, R_r,obs_ is the rate of rest break observed in +6°C, and T_upp_ is the upper threshold temperature.

For all the parameters in the overall model other than T_upp_ and t_0_ (start of the rest period), we obtained an estimate based on explicit experimental data from our study. Using those estimates in test simulations, we estimated the values of T_upp_ and t_0_ by minimizing the root mean square error (RMSE) between the predicted and the observed timing of bud burst in the nine treatments of the WTC experiment. For t_0_, we tested three values: November 1, November 15, and November 23. For T_upp_, we tested 15 values in the range of +4 to +20°C, with a step of 1°C.

#### Sub-Model II

For any constant temperature T′, the empirical value of the potential rate of ontogenetic development was calculated as (Sarvas, 1972; Campbell, 1978; Zhang et al., 2021c)


(7) $$R_{o,pot}\left(T^{\prime }\right)=100\frac{1}{DBB\left(T^{\prime }\right)}$$

where, DBB = days to bud burst (leaf-out) required under the experimental temperature T′.

As a result of using the multiplier 100, the scale for the ontogenetic development is the percentage scale [0,100%], so the value of R_o,pot_ calculated with Equation (7) indicates the percentage of ontogenetic development, out of the total ontogenetic development required for leaf-out, taking place in a day. The value of DBB, initially expressed in terms of days, was converted to hours by multiplying the original value by 24, because the model was applied with a time step of 1 h.

The spring experiment produced the DBB data for Equation (7) should be started as close as possible to the time of rest completion under natural conditions (refer to section “Constructing the sub-models on the basis of experimental data” above). The time of rest completion under natural conditions was estimated in the autumn experiment using a chilling treatment under outdoor natural conditions. In that treatment, the DBB curve did not show leveling off even in the latest transfers from the chilling to the forcing conditions. Accordingly, following Zhang et al. (2021c), we determined the Δt for the natural chilling treatment as follows: first, equation (2) was fitted to the scatter plot in a way similar to that used with the controlled chilling data. Using the fitted curve, the value of Δt was determined as the duration of chilling corresponding to the same value of DBB as was obtained with the +6°C treatment by the leveling off of the DBB curve (refer to Figure 1D in the section “Results”). The starting date of the first transfer (February 13) was the closest to the estimated time of rest completion under natural conditions so the results of the first transfer were used for constructing sub-model II (refer to section “Results”).

Using Equation (7), the DBB values obtained for the individual seedlings in the first transfer of the spring experiment were first converted into the corresponding values of R_o,pot_. The R_o,pot_ values, thus, obtained were then plotted against the experimental temperature, and the following sigmoidal equation was fitted to the scatter plot (Hänninen, 1990; Zhang et al., 2021c):


(8) $$R_{o,pot}\left(T\right)=\frac{a_{2}}{1+e^{\frac{1}{b_{2}}\left(T-c_{2}\right)}}$$

where, T(t) is the air temperature, and a_2_, b_2_, and c_2_ are parameters determining the upper asymptote, the slope, and the inflexion point of the sigmoidal curve, respectively.

#### Sub-Model III

In a chilling-forcing experiment, the empirical value of ontogenetic competence, C_o_, for any given duration of chilling, x days, is obtained by definition as the ratio of DBB at rest completion to its value at x days of chilling: C_o_(x) = DBB(Δt)/DBB(x) (Zhang et al., 2021c). However, this mathematics assumes that all seedlings show bud burst, so BB% = 100%. As in several earlier studies, this was not the case in our study; so, in order to account for the seedlings not showing bud burst after short durations of chilling, we set the value of C_o_ to zero with chilling durations that showed a BB% value below 50%. In all, then, the empirical value of C_o_ for a given chilling duration x was calculated as follows (Zhang et al., 2021c):


$$C_{o}\left(x\right)=0,whenBB\%\left(x\right)<50\%$$

(9) $$C_{o}\left(x\right)=\frac{f_{1}\left(\Delta t\right)}{f_{1}\left(x\right)},whenBB\%\left(x\right)\geq 50\%$$

where, f_1_ is the function from equation (2).

In order to formulate sub-model III, the empirical values of C_o_(x) obtained by means of Equation (9) were plotted against the corresponding values of the state of rest break, S_r_. The latter were calculated by integrating the rate of rest break, calculated with Equation (6), up to the end of day x. Subsequently, a piece-wise linear model was fitted to the data (Zhang et al., 2021c):


$$C_{o}\left(S_{r}\right)=0,S_{r}<S_{r,min}$$

(10) $$C_{o}\left(S_{r}\right)=a_{3}S_{r}+1-100a_{3},S_{r}\geq S_{r,min}$$

### Model Calculations

#### Procedures Applied in All Calculations

All calculations with the process-based tree phenology model developed were started on t_0_ = November 15 (for estimating the value of t_0_, refer to section “Results”). The model was run on an hourly basis using hourly temperature data as input for calculating the hourly values of R_r_(t) (Equation 6) and R_o,pot_(t) (Equation 8). The rate of ontogenetic development, R_o_(t), was calculated by multiplying R_o,pot_(t) by the ontogenetic competence, C_o_(t) (Supplementary Figure 1). The integrations for S_r_(t) and S_o_(t) were carried out numerically by summing up the hourly values of R_r_(t) and R_o_(t). The value of S_r_(t) was kept at 100% for the annual cycle being simulated once that value, indicating rest completion, was attained. In calculations involving several years, a new annual cycle was started on the next t_0_ = November 15 by setting S_r_(t) = 0% and S_o_(t) = 0% once leaf-out had been predicted (S_o_(t) = 100%).

All model calculations and statistical analyses were conducted with R studio (R version 4.0.0; R Core Team, 2020).

#### Tests of the Model and Analyses of the Dormancy Dynamics Predicted by It

As stated above, the values of the parameters t_0_ and T_upp_ were estimated by fitting the overall model to the air temperature measurements and leaf-out observations in the WTC experiment. At the same time, the accuracy of the model was examined by evaluating the RMSE, but due to the estimation of the values of the two parameters, this examination did not provide an entirely independent test of the accuracy of the model (internal validation).

Using the natural-temperature data collected in the outdoor seedling collection on our campus, the dormancy dynamics predicted by the model was illustrated by calculating and plotting the values of the state of rest break, S_r_, and the state of ontogenetic development, S_o_, for November 15, 2017, to the predicted time of leaf-out in spring 2018. The predicted timing of rest completion was compared with the one observed in the experiment with natural chilling (for the latter, refer to Figure 1D in the section “Results”). Correspondingly, the predicted timing of leaf-out was compared with the one observed in the outdoor seedling collection. Observations of the seedlings under the outdoor chilling conditions were not used in formulating the model so this prediction provided an independent yet single test of the overall model.

#### Scenario Analyses

We applied the process-based model developed in this study to scenario analyses where the effects of climatic warming were projected on (1) the timing of the leaf-out of the pecan seedlings and (2) the subsequent risk of cold damage to them. The scenario simulations were carried out over the period 2022–2099 for the three locations: our research site Hangzhou, Hefei (31°51″N,117°10″E), and Nanping (27°20″N,118°7″E). Hangzhou and Hefei are located in the main pecan cultivation area in subtropical southeastern China, whereas pecan cultivation is less common at the southern location Nanping (Zhang et al., 2015).

For each location, the projections were calculated using the daily minimum and maximum temperatures from the warming scenarios RCP4.5 and RCP8.5 (Meinshausen et al., 2011; Thrasher et al., 2012). For Hangzhou, Hefei, and Nanping, they represented warming by 1.4 (4.8°C), 1.7 (5°C), and 1.8°C (4.1°C), respectively (The values in parentheses are those for RCP8.5). The hourly temperature values needed in the simulations were generated from the daily minimum and maximum temperatures following Zohner et al. (2020). For both RCP4.5 and RCP8.5, the projected leaf-out dates, expressed as DOY values, were plotted against the simulation year, and a linear regression line, indicating the rate of advancing or delay of leaf-out over 2022–2199, was fitted to the scatter plot of each warming scenario. In the fitting of the linear regression lines, it was assumed that the two lines representing the two warming scenarios for a given location had the same value for the first simulation year 2022. The value was calculated as the average of the DOY values projected for the two respective scenarios for the location. The interception parameter was fixed to meet this criterion. Subsequently, the slope parameter was estimated by fitting the line to the observations.

In order to examine the risk of damage caused by low temperatures (false spring risk), the occurrence of cold damage events was determined by examining whether the air temperature dropped below a critical threshold, T_dam_, of the damaging temperature range during a time window of 10 days before the projected leaf-out to May 31. In the absence of explicit data for T_dam_, the threshold T_dam_ = +5°C was determined on the basis of earlier indicative results (Kaur et al., 2020) and on the basis of recent experiences from a pecan orchard (Supplementary Figure 3). The cold damage was quantified for each year by its incidence (number of damaging days) and severity (the lowest temperature occurring during the day or days). Finally, the cold damage year percentage (CDY%) for the whole simulation was determined as the percentage of the years with projected cold damage out of the 79 years simulated.

We had no explicit data for determining the value of the parameter T_upp_. That is why a sensitivity analysis was carried out by calculating the CDY% values for four additional values of T_upp_, in addition to the estimated value of T_upp_ = +13°C (refer to Figure 4 in the section “Results”). The tested T_upp_ values ranged from +11 to +15°C, with a step of 1°C.

## Results

### Experimental Results and the Resulting Process-Based Leaf-Out Model

**FIGURE 2:**
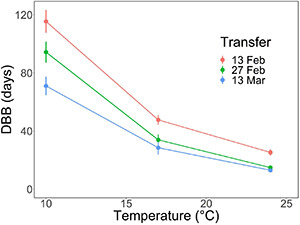
Effects of air temperature on days to bud burst, DBB (mean ± SE), of first-year pecan seedlings in the spring experiment. The transfer dates indicate the time when the seedlings were transferred from the outdoor natural overwintering conditions to the three growth chambers with respective forcing temperatures of +10, +17, and +24°C. Note that the phenological event observed was leaf-out, but that the concept “bud burst” is used here as a generic one facilitating the use of the standard index DBB.

With both natural and controlled chilling, the bud burst percentage, BB%, increased significantly from 10% at zero days of chilling to 100% in the fourth transfer after 42 days of chilling (Figures 1A,B and Table 1). The days to bud burst, DBB, decreased significantly with prolonged chilling, both natural and controlled (Table 1). For the controlled chilling, the decrease was clearly exponential, showing leveling off with long durations of chilling (Figure 1C), whereas for natural chilling, the decrease in DBB with prolonged chilling was only slightly exponential, with no clear leveling off (Figure 1D). In the spring experiment, DBB decreased significantly with increased forcing temperature (Figure 2 and Table 1). Furthermore, DBB also decreased significantly from the first transfer on February 13 to the last one on March 13 (Figure 2 and Table 1).

**TABLE 1:** Statistical analysis of factors affecting the days to bud burst, DBB, and the bud burst percentage, BB%, in the autumn and spring experiments (BB% in the autumn experiment only).

**Experiment**	**Factor**	***DBB***		***BB%***
		***F***	***P***	***P***
Autumn experiment	Chilling type	**27.7459**	**< 0.001**	0.993
	Transfer	**38.9620**	**< 0.001**	**< 0.001**
	Transfer*Chilling type	1.6253	0.642	0.955
	Transfer	**23.446**	**< 0.001**	
Spring experiment	Temperature	**189.461**	**< 0.001**	
	Transfer*Temperature	**3.014**	**0.023**	

*A two-way ANOVA was applied to the analysis of the DBB results and logistic regression with a binary response of factors to the analysis of the BB% results. In the autumn experiment, the chilling type refers to the difference between chilling under controlled conditions at +6°C and chilling under natural outdoor conditions. The *F* and *P* values in bold indicate statistical significances with at least *P* < 0.05.*

**FIGURE 3:**
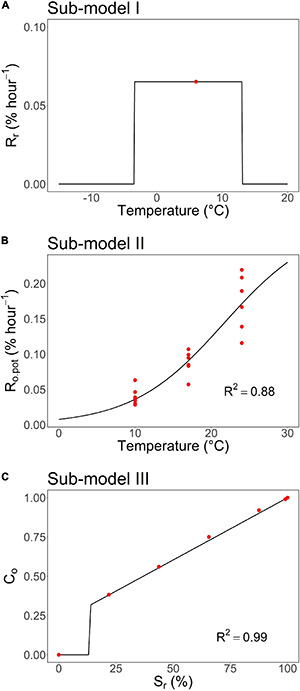
The three sub-models constitute the process-based leaf-out phenology model developed in this study for first-year pecan seedlings. **(A)** Sub-model I: air temperature response of the rate of a rest break, R_r_, (Equation 6). **(B)** Sub-model II: air temperature response of the potential rate of ontogenetic development, R_o,pot_, (Equation 8). **(C)** Sub-model III: dependence of ontogenetic competence, C_o_, on the state of rest break, S_r_, (Equation 10). For the structure of the overall model and the relationships between the sub-models, refer to Supplementary Figure 1. The red dots represent the empirical data points calculated based on results from the autumn and spring experiments (Figures 1, 2) and the black lines and curves the response functions fitted to the data points. For the equations and details of constructing the models, refer to “Constructing the sub-models on the basis of experimental data” in the section “Materials and Methods.” The parameter values of the models can be found in Table 2.

For constructing sub-model I, the value of Δt = 64 days was obtained in the autumn experiment for the duration of chilling required for rest break in the controlled chilling at constant +6°C (Figure 1C). On an hourly basis, this implies an empirical rate of rest break R_r,obs_ = 100%/(64 days × 24 h day^–1^) = .065% h^–1^ at +6°C (Equation 1; Figure 3A and Table 2).

**TABLE 2:** Parameter values of the process-based leaf-out phenology model developed in this study for first-year seedlings of subtropical pecan.

**Sub model**	**Parameter**	**Value**
Overall model	t_0_	15 November
Sub-model I	T_upp_	+13°C
	R_r,obs_	0.065% per hour
Sub-model II	a_2_	0.287
	b_2_	−5.999
	c_2_	21.64
Sub-model III	a_3_	0.0079279
	S_r,min_	14%

*Sub-model I: air temperature response of the rate of rest break, R_*r*_ (Equation 6, Figure 3A). Sub-model II: air temperature response of the potential rate of ontogenetic development, R_*o,pot*_(Equation 8, Figure 3B). Sub-model III: dependence of ontogenetic competence, C_*o*_, on the state of rest break, S_*r*_ (Equation 10, Figure 3C). For the structure of the overall model, refer to Supplementary Figure 1.*

**FIGURE 4:**
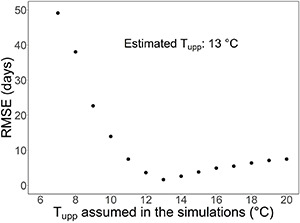
Estimation of the upper threshold of the rest-breaking temperature range, T_upp_ (refer to Figure 3A) by fitting the overall model to the results of the nine temperature treatments of the whole-tree chamber (WTC) experiment. The graph shows the root mean square error (RMSE) of the leaf-out timing predicted by the overall model for the experiment as a function of the value of T_upp_ used in the simulations. With each value of T_upp_, the calculations were started on t_0_ = November 15, because the use of that date minimized the RMSE among the dates tested (refer to the section “Results” in the text). With the lowest value of T_upp_ tested (+6°C), the model failed to predict any leaf-out for three of the nine temperature treatments in the experiment because of insufficient chilling. This is why no RMSE is given for T_upp_ = +6°C.

The upper threshold, T_upp_, of the rest-breaking temperature range in sub-model I was estimated by fitting the overall model (Supplementary Figures 1, 3) to the leaf-out observations in the WTC experiment (Figure 4). Simultaneously, the value of parameter t_0_, starting day of rest period, was estimated by carrying out the calculations for estimating T_upp_ with three different values of t_0_. The values of RMSE obtained for the three t_0_ values November 1, November 15, and November 23 were 2.11, 1.67, and 2.24 days, respectively, implying the value of 15 November for t_0_ (Table 2). Using that value of t_0_, the RMSE between the predicted and the observed timing of leaf-out was minimized with T_upp_ = +13°C (Figure 4); so, that value was adopted as the estimate of T_upp_ (Table 2).

Sub-model II addresses the air temperature response of the potential rate of ontogenetic development, R_o,pot_. This implies that the experiment for determining sub-model II should be started as close as possible to the estimated time of rest completion under natural conditions (refer to “Constructing the sub-models on the basis of experimental data” in the section “Materials and Methods”). In the autumn experiment, rest completion under natural conditions was estimated to take place at Δt = 90 days after the onset of the experiment on November 21, 2017 (Figure 1D), i.e., February 19, 2018. This means that the first (February 13), second (February 27), and third transfers (March 13) in the spring experiment (Figure 2) were carried out 6 days before, 8 days after, and 22 days after the estimated rest completion, respectively. Accordingly, the results of the first transfer in the spring experiment (Figure 2) were used in the construction of sub-model II (Figure 3B and Table 2).

The empirical value of ontogenetic competence, C_o_, was zero in the first transfer with S_r_ = 0% (Figure 3C). This was because of the low value of BB% in the first transfer (Figure 1A; refer to Equation 9). Starting from the second transfer, the empirical value of C_o_ increased with increasing S_r_ (= with prolonged chilling). Accordingly, a piece-wise linear relationship with a discontinuity at S_r,min_ = 14% was determined for sub-model III (Figure 3C and Table 2).

### Model Test and Predictions

According to the model prediction for the dormancy dynamics for the period of autumn 2017 to spring 2018, rest break started immediately at the beginning of the simulation in November 2017, as shown by the increasing value of the state of rest break, S_r_, at that time (Supplementary Figure 4B). This was because the air temperature (Supplementary Figure 4A) at the time fluctuated mainly in the range causing rest break (Figure 3A). The simulated rest break progressed at an almost constant rate, leading to predicted rest completion on February 1, 2019, 20 days before the independently observed rest completion on February 21 (Supplementary Figure 4B), estimated on the basis of the autumn experiment results (Figure 1D). No ontogenetic development was predicted for the beginning of the simulation, as shown by the values of S_o_ = 0% at that time (Supplementary Figure 4B). That was because with S_r_ < 14%, the ontogenetic competence stayed at C_o_ = 0 (Figure 3C; ontogenetic competence not shown in Supplementary Figure 4B). With further rest break beyond S_r_ = 14%, ontogenetic development also started, but the S_o_ increased slowly at first because the ontogenetic competence was still relatively low and the air temperatures (Supplementary Figure 4A) were generally too low to promote ontogenetic development (refer to Figure 3B). Toward spring, the predicted rate of ontogenetic development increased, and leaf-out was predicted to occur on April 2, 2018, 1 day after leaf-out was independently observed under natural conditions (Supplementary Figure 4B).

**FIGURE 5:**
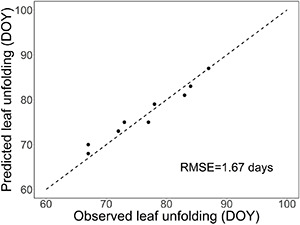
Test of the process-based leaf-out phenology model developed in this study for first-year pecan seedlings by leaf-out observations from a WTC experiment with nine temperature treatments.

The results presented in Supplementary Figure 4 provide an independent test of our model with observational data. However, as only 1 year was included in the test, the test is relatively weak, but it serves to illustrate the dormancy dynamics predicted by our process-based leaf-out model. In the main test, the model simulated the timing of leaf-out in the nine treatments of the WTC experiment with good accuracy (RMSE = 1.67 days, Figure 5).

### Projections of Leaf-Out Timing and Cold Damage for Climatic Warming

**FIGURE 6:**
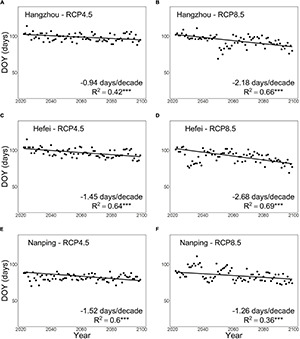
Projected timing of leaf-out in first-year pecan seedlings in three locations in subtropical southeastern China from 2021 to 2099. Using the process-based phenology model developed in this study, the leaf-out was projected for the warming scenarios RCP4.5 (panels on the left) and RCP8.5 (panels on the right) for **(A,B)** Hangzhou and **(C,D)** Hefei, located in the main pecan cultivation area in the northern part of the subtropical monsoon climate zone, and for **(E,F)** Nanping, located south of the main pecan cultivation area. “***” indicates significance at *P* < 0.001.

For both RCP4.5 and RCP8.5, our model projected an overall advancing trend in pecan seedling leaf-out in all three locations addressed (Figure 6). There were, however, considerable differences between the locations and the warming scenarios. For the two northern locations Hangzhou and Hefei, an overall advancement at rates of about −1 and −1.5 days per decade, respectively, was projected under RCP4.5 (Figures 6A,C), and the rate was about double under RCP8.5 (Figures 6B,D). In the southern location Nanping, the advancement rate was about −1.5 days per decade under RCP4.5 and slightly smaller (less negative) under RCP8.5 (Figures 6E,F). In all three locations, there was more year-to-year variation in the timing of leaf-out under RCP8.5 than under RCP4.5 (Figure 6). In the southern location Nanping, the large year-to-year variation led to a delay in leaf-out in many years during the first decades of the 21st century despite the overall advancement trend (Figure 6F).

**FIGURE 7:**
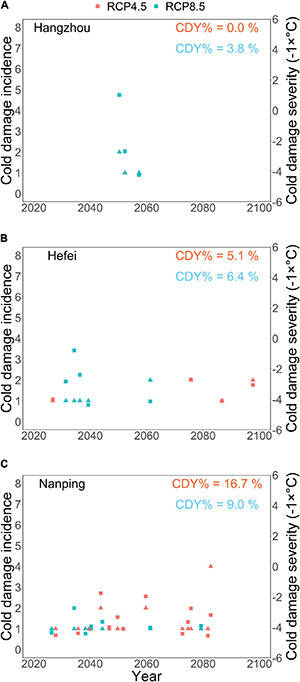
Projected cold damage to the first-year pecan seedlings in three locations in subtropical southeastern China from 2021 to 2099 under the warming scenarios RCP4.5 (red symbols) and RCP8.5 (blue symbols).**(A)** Hangzhou and **(B)** Hefei are located in the main pecan cultivation area in the northern part of the subtropical monsoon climate zone and **(C)** Nanping south of the main pecan cultivation area. The cold damage was projected by examining whether the air temperature dropped below the threshold of +5°C during the time window from 10 days before the projected leaf-out (Figure 6) to May 31. If cold damage was projected for a given year, it was quantified by its incidence (number of days when damaging air temperatures occurred: triangles, vertical axis on the left-hand side), and severity (the lowest temperature that occurred during those days: squares, vertical axis on the right-hand side). In order to make both cold damage indices increase from the bottom to the top of the panels, the severity is expressed by the opposite number of the corresponding temperature. The values of CDY% in red and blue indicate the percentage of cold damage years for the respective warming scenarios out of the 79 years simulated.

Our model projected no cold damage for Hangzhou under RCP4.5 (cold damage year percentage CDY% = 0), and under RCP8.5, projected cold damage was rare (CDY% = 3.8) (Figure 7A). Projected cold damage was also quite rare in Hefei, with the CDY% values at 5.1 and 6.4 for RCP4.5 and RCP8.5, respectively (Figure 7B). For Nanping, much more frequent cold damage was projected, and contrary to the two northern locations, there was more projected cold damage under RCP4.5 (CDY% = 16.7) than under RCP8.5 (CDY% = 9) (Figure 7C). No clear differences among the three locations were found in the annual indices of cold damage (incidence and severity), with the following two exceptions: first, in the simulation for Nanping under RCP4.5, there was one exceptional year with an exceptionally high incidence (4 days) of cold damage (Figure 7C). This is in line with the projection of cold damage being common in Nanping (CDY% = 16.7) under RCP4.5. Second, even though cold damage was projected for only 3 years in Hangzhou under RCP8.5 (CDY% = 3.8), it was more severe in Hangzhou than in the other two locations in one of those years (Figure 7).

In the sensitivity analysis, only a minor effect of the parameter T_upp_ on the CDY% values was found for Hangzhou and Hefei, indicating that our results for these two locations are robust (Supplementary Table 1). For Nanping, however, low values of T_upp_ (+11 and +12°C) decreased the CDY%, and high values of T_upp_ (+14 and +15°C) increased it in comparison with the CDY% values obtained in the main simulation with the estimated value of T_upp_ = +13°C (Supplementary Table 1).

## Discussion

### Biological Realism of the Process-Based Tree Phenology Models

Sparks (1993) demonstrated experimentally the existence of rest period (endodormancy) and chilling requirement for pecan trees growing over a large geographical range in the United States. Our experimental findings confirm that seedlings of the subtropical pecan grown in southeastern China also evince these traits. This is in line with recent studies, which show that several subtropical tree species have a rest period and a chilling requirement (Du et al., 2019; Song et al., 2020; Pan et al., 2021; Zhang et al., 2021a, b). The novelty and scarcity of these findings in the case of subtropical trees are in striking contrast with the case of boreal and temperate trees, where the existence of these traits has already been known for a 100 years (Coville, 1920; Jewaria et al., 2021).

We introduced a process-based spring leaf-out model for the subtropical first-year pecan seedlings we examined in this study. Our main emphasis was on the biological realism of the models (Levins, 1966, 1968; Charrier et al., 2015; Hänninen, 2016), which is why the model development was based on a hypothetico-deductive (HDM) approach, which makes use of experiments explicitly designed for examining the responses modeled (sub-models I–III). The approach is regarded as an HDM one because the model development is based on the implications of the models for the occurrence and timing of the readily observable leaf-out under experimental conditions rather than direct observations and measurements of the modelled physiological and morphological phenomena of rest break and ontogenetic development. Accordingly, all the data points in Figure 3 are determined by analyzing the leaf-out data gathered under the experimental conditions.

This approach, referred to as the ecophysiological approach by Hänninen (2016), has been taken in process-based tree phenology modeling only rarely (Sarvas, 1972, 1974; Campbell and Sugano, 1975, 1979; Caffarra et al., 2011; Zhang et al., 2021c). This is obviously because the experiments are time-consuming and laborious. Most earlier studies with process-based tree phenology modeling have been based on the technique of inverse modeling, where the model parameters are estimated by fitting the model to observational long-term phenological and air temperature records (Kramer, 1994; Chuine et al., 1998; Basler, 2016; this is a phenological approach in the terminology of Hänninen, 2016).

This is unfortunate, for it has been known since the pioneering study of Hunter and Lechowicz (1992) that inverse modeling with observational data runs a high risk of producing biologically unrealistic process-based models of tree phenology. To take just two different examples of the problems related to inverse modeling (for a review of the problems, refer to Hänninen et al., 2019), first, by applying inverse modeling to the growth onset of Scots pine (*Pinus sylvestris*) saplings in a warming experiment, Hänninen (1995) found that a model based on photoperiod outcompeted all models in which chilling causes rest break. However, in an explicit experiment where photoperiod was controlled, he found that photoperiod does not affect the rest break of Scots pine saplings at all. The cause of the problem was that in the warming experiment the natural photoperiod correlated with chilling accumulation, which actually caused the rest break. Second, using an inverse modeling approach, Luedeling et al. (2021) recently found that temperatures of up to +30°C cause rest breaks in apples (*Malus domestica*). As discussed by the authors themselves, this result is most probably an artifact caused by the lack of temperatures above +10°C in the observational data set.

Despite the experimental approach taken, our model still needs further testing and development. This is especially true in the case of sub-model I, where we had just one empirical data point for the model. This is where our study deviated most from the study of Zhang et al. (2021c), which we otherwise followed closely in building the model on the basis of experimental data. We solved the problem following Lundell et al. (2020), who also had just one controlled chilling temperature in their experiment: (1) the lower threshold of the rest-breaking temperature range was set at −3.4°C (Sarvas, 1974), (2) the upper threshold T_upp_ was estimated using an inverse modeling approach of fitting the model to the experimental results, and (3) a plateau response was assumed between the two threshold temperatures. In this way, we obtained a reasonable fit of the overall model to the experimental results (Figure 5). It should also be noted that in the mainline inverse modeling approach that uses observational phenological records (Kramer, 1994; Chuine et al., 1998; Basler, 2016), there are no experimentally determined data points available for estimating the value of any parameter of any sub-model.

Our estimated value of T_upp_ = +13°C is higher than the upper thresholds usually applied in process-based models for boreal and temperate trees (Sarvas, 1974; Cannell and Smith, 1983; Wang et al., 2020; Xu et al., 2020). However, the value is well in line with the experimental results available for subtropical trees. Zhang et al. (2021a, c) found that in two subtropical tree species the rest-breaking effect of +15°C was equal to that of +10 and +5°C, suggesting that in subtropical trees T_upp_ can even be well above +15°C (refer to also Jewaria et al., 2021). Further experimental studies with several controlled chilling temperatures and chilling durations (Baumgarten et al., 2021; Zhang et al., 2021a, c) are needed to test and improve the sub-model I developed for pecan seedlings in this study.

The starting date of the rest period (endodormancy) used in this study (t_0_ = November 15) also involves a great deal of uncertainty. In that respect, our model is no exception among the published process-based tree phenology models. To the best of our knowledge, the value of t_0_ is not based on explicit ecophysiological data in any of them (but refer to Fuchigami et al., 1982 and the related discussion in Hänninen, 2016, p. 119–120). Arbitrary starting dates, such as September 1 (Hänninen, 1990, 1991; Chuine et al., 1998, 2016) and November 1 (Cannell and Smith, 1983; Kramer, 1994), have been used with boreal and temperate trees earlier on. In subtropical trees, bud set typically occurs from September to October (Zhang et al., 2021a), which is why we used November 1 as the first candidate when estimating the value of t_0_.

For our experiments, we used seedlings raised from seeds collected in the seminatural pecan forests of our study area in the Hangzhou region. It is well-known that seminatural pecan trees are of subtropical origin, but no exact information about their genetic background is available (Zhang et al., 2015). In pecan nut prediction, bred cultivars are normally used, which implies that the generality of our model should also be tested with bred cultivars in future studies.

### Projected Effects of Climatic Warming

Depending on the geographical location, the warming scenario, and the year of simulation, all three of our hypotheses gained support from some part of our simulation results. This even held for the delay hypothesis, for in many years of the first decades of the 21st century, leaf-out was delayed in the simulations for the southern location Nanping under the RCP8.5 warming scenario, despite the overall advancement trend found for Nanping under RCP8.5. For most years, however, advancement of leaf-out was projected for all the three locations and under both climatic scenarios addressed. In comparison with earlier studies with temperate trees (Kramer, 1994; Murray et al., 1994; Wang et al., 2020), the advancement rates found in this study were relatively low. This suggests that reduced chilling restricts the advancement of spring phenology more under the warm subtropical conditions than under the cooler temperate conditions.

A clear although not very rapid advancement of leaf-out was projected for the two northern locations, Hangzhou and Hefei. This held for both RCP4.5 and RCP8.5, and the rate of advancement under RCP8.5 was about double the rate under RCP4.5. These results show that in Hangzhou and Hefei, the effect of increased forcing overrode the effect of decreased chilling on the projected timing of leaf-out. For these two locations, cold damage was projected quite infrequently: once every 26 years (CDY% = 3.8%) at most in Hangzhou and once every 16 years (CDY% = 6.4%) in Hefei. In all, despite some cold damage projected, the prolonged growing season hypothesis was mainly supported by our findings for Hangzhou and Hefei.

The results projected for the southern location Nanping deviated from those found for the two northern locations in many ways. First, the advancement rate of leaf-out in Nanping was almost identical for the two warming scenarios. This showed that the effect of reduced chilling was stronger effect under the warming of the warmer climate in Nanping than under the warming of the cooler climates of the two northern locations and that Nanping is ultimately receiving less chilling with warming, which is impacting leaf-out timing. Second, as the projected advancement rates for Nanping were quite similar under the two climatic scenarios, more cold damage was projected under RCP4.5 than under the warmer scenario of RCP8.5. In Nanping, the value of CDY% was 16.7 for RCP4.5 and 9 for RCP8.5, implying that cold damage would occur once every 6 (RCP4.5) or once every 11 (RCP8.5) years. However, the results of the sensitivity analysis show a higher uncertainty for Nanping than for the other two locations. For Nanping, much less cold damage was projected for the low values (+11 and +12°C) of T_upp_ than for the estimated value of it (T_upp_ = +13°C) (Supplementary Table 1). This finding emphasizes the need for further experimental studies to improve sub-model I (refer to above).

In Nanping, CDY% was exceptionally higher under the cooler scenario RCP4.5 than under the warmer scenario RCP8.5. This was caused by the similar timing of leaf-out projected in Nanping for the two climatic scenarios. In other words, if leaf-out occurs simultaneously in a cooler and a warmer climate, then probably there is more cold damage in the cooler than in the warmer climate. For Nanping, then, a paradoxical conclusion arises: warming as such causes cold damage by false springs, but rapid warming causes such damage less than slow warming because with rapid warming the climate gets so warm after some decades that the potentially damaging temperatures below +5°C do not occur around the time of leaf-out any longer. This is why the projected damage in Nanping under RCP8.5 concentrates on the early decades, whereas under RCP4.5 cold damage is also projected for the late years. A similar phenomenon was actually found for Hefei; however, the results for Hefei were different in other respects (refer to the previous paragraph). This finding suggests that in the southern part of the subtropical zone, where the climate is relatively warm already, rapid warming creates a time window of high risks of false springs for a few decades. After the time window closes, the risk of cold damage decreases.

The modeling approach adopted in this study has been applied earlier for assessing the effects of climatic warming on spring phenology and the risk of false springs in various boreal and temperate trees (Cannell, 1985; Hänninen, 1991; Kramer, 1994; Linkosalo et al., 2000). The approach holds great potential for assessing the suitability of perennial horticultural crops for cultivation under climatic warming at different geographical locations. For instance, under climatic warming, many subtropical crops may find new cultivation areas under temperate conditions. The present modeling approach is well-suited for assessing the suitability of subtropical crops to such transfers to temperate conditions.

## Conclusion

We introduced a process-based phenology model for the leaf-out of pecan seedlings grown in subtropical southeastern China. In order to maximize the biological realism of the model, we based its development on experiments explicitly addressing the responses of the developmental phenomena modeled. The model was applied to project the timing of leaf-out and risk of false spring in two locations in the current main pecan cultivation area in the northern part of the subtropical monsoon climate zone in southeastern China and one location south of the main cultivation area. An overall advancement of leaf-out was projected for all three locations. For the two northern locations in the main cultivation area, no considerable increase in cold damage caused by false springs was projected. This finding suggests that pecan cultivation can continue relatively safely in these locations in the upcoming decades, too, even though under the RCP8.5 warming scenario, damage caused by false spring was projected to occur once every 16–26 years in these two northern locations, too. Paradoxically, cold damage caused by false springs was projected to occur more frequently in the southern location, once every 6 and 11 years under the warming scenarios RCP4.5 and RCP8.5, respectively. Due to the uncertainties related to the air temperature response of rest break (endodormancy release) to chilling, our conclusions need to be further addressed in further experimental studies and model development based on it. Similarly, the uncertainties related to the genetic background of our research material should be addressed by testing our model with pecan cultivars used in commercial nut production. On the basis of our present results, we do not recommend starting large-scale pecan cultivation in locations south of the present main pecan cultivation area in southeastern subtropical China.

## Data Availability Statement

The raw data supporting the conclusions of this article will be made available by the authors, without undue reservation.

## Author Contributions

RZ designed the study. JZ, JL, and SS carried out the experiments. JZ, RZ, and HH analyzed the data and carried out the modeling. JZ, HH, and RZ wrote the manuscript. All authors contributed to the article and approved the submitted version.

## Conflict of Interest

The authors declare that the research was conducted in the absence of any commercial or financial relationships that could be construed as a potential conflict of interest.

## Publisher’s Note

All claims expressed in this article are solely those of the authors and do not necessarily represent those of their affiliated organizations, or those of the publisher, the editors and the reviewers. Any product that may be evaluated in this article, or claim that may be made by its manufacturer, is not guaranteed or endorsed by the publisher.
