# Supplementary material for: Extending the Cultivation Area of Pecan (Carya illinoinensis) Toward the South in Southeastern Subtropical China May Cause Increased Cold Damage
Source: Front Plant Sci. 2021 Nov 30;12:768963. doi: 10.3389/fpls.2021.768963 (PMC8669331; doi:10.3389/fpls.2021.768963)
Supplement: Supplementary file 1 [file Data_Sheet_1.pdf]

SUPPLEMENTARY MATERIAL

MATERIALS AND METHODS

Experimental Material

We conducted the experiments on Zhejiang A&F University campus (30°15″N, 119°43″E), located in south-eastern China on the northern edge of the subtropical monsoon climate zone. The mean annual precipitation in the area is 1614 mm. The mean annual air temperature is +15.6 °C, and the mean monthly air temperature varies from +4.5 ºC in January to +28.9 ºC in July (Zhang, 2015).

Pecan (*Carya illinoinensis*) was introduced into subtropical China as seeds from the USA about a hundred years ago. Seeds of subtropical pecan provenances were used in nurseries in south-eastern China to produce the current seminatural stands in subtropical China (Zhang et al., 2015). For the three experiments carried out in the present study, we used first-year pecan seedlings raised from seeds collected from the seminatural stands (**Figure S2**). The autumn and spring experiments in 2017 – 2018 were designed to determine the effects of air temperature on rest break and ontogenetic development, respectively. The information obtained was used for constructing the process-based leaf-out model. To test the model, a whole-tree chamber (WTC) experiment was carried out in 2019 – 2020. The WTC experiment was also used for estimating two of the model parameters.

For the autumn and spring experiments in 2017 and 2018, containerized pecan seedlings were produced in a commercial nursery in Nanjing (31°57´N, 118°51´E) with standard nursery practices. In brief, open pollinated seeds were collected from native seminatural stands in early November 2016 and were then air-dried for seven days. The seeds were stored at +5 ºC in a refrigerator until sowing time. In early March 2017 the seeds were sown in a greenhouse (+25±5 ºC) into a growth medium consisting of 5 peat : 3 vermiculite : 2 perlite by volume (Shanghai Jizhi Agricultural Science and Technology Co., Shanghai, China). In early May, when the developed seedlings had three to five leaves, the seedlings were transplanted into polyethylene pots with a diameter of 12 cm and height of 17 cm with the same growth medium as before. The containerized seedlings were then grown in the field and were irrigated with sprinklers to keep the growth medium well watered. Weeds were removed by hand as necessary.

The seedlings were transferred to the university campus on 29 October 2017 and were kept outdoors until the beginning of the two respective experiments in autumn 2017 and spring 2018. Natural defoliation occurred in early to mid-November. For the WTC experiment in 2019 – 2020, containerized first-year pecan seedlings were produced in a commercial nursery in Lin'an (30°41″N 119°37″E) with standard nursery practices similar to those used earlier in the Nanjing nursery for producing the seedlings for autumn and spring experiments of 2017 and 2018. The seedlings were transferred to the university campus on 15 November 2019 and were kept outdoors until the beginning of

the experiment in November. Natural defoliation had occurred prior to the transfer to the campus.

Experimental Practices Shared by All the Experiments

For the determination of leaf-out, the terminal bud of the seedlings was observed, and the following four developmental stages were discerned: bud closed, bud swelling, leaf emergence, and leaf-out. The first three stages were observed in order to improve the precision of the fourth stage, leaf-out, which was the observation used in the analysis of the results (Zhang et al., 2021a). In the growth chambers, weeds were removed by hand as necessary, and the seedlings were irrigated regularly to keep the soil in the pots moist. Hourly air temperatures were recorded throughout the experiments in all growing conditions by two or three iButton thermometers (Model DS1912L, Embedded Data Systems Co., Ltd, KY, USA).

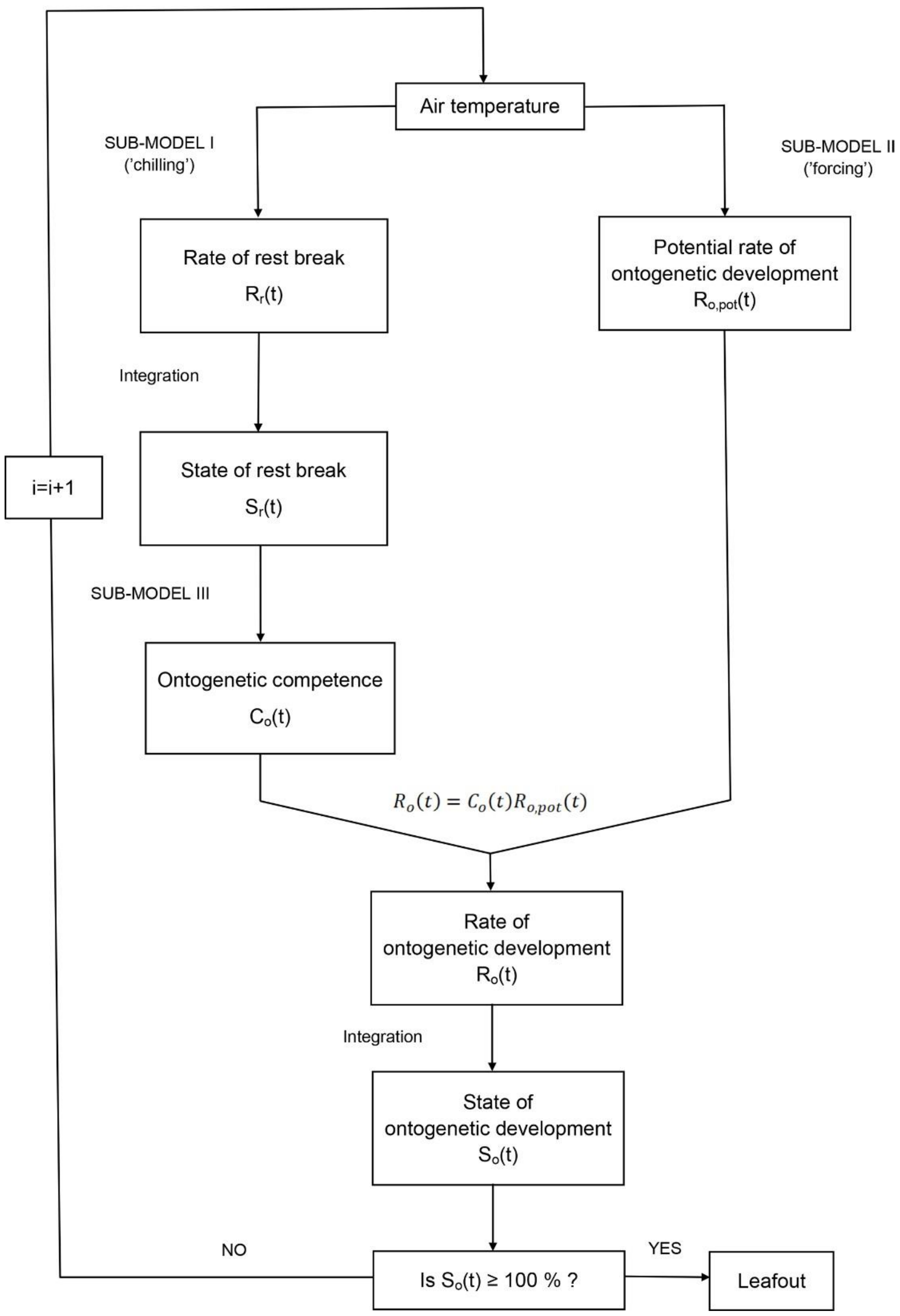

FIGURE S1. Modular structure of the process-based model developed in the present study for leaf-out phenology in first-year pecan seedlings. For details, see section 'Structure of the overall model' in Materials and methods. (Modified from Hänninen 1990, 2016; Hänninen and Kramer, 2007; Lundell et al., 2020; Zhang et al., 2021b).

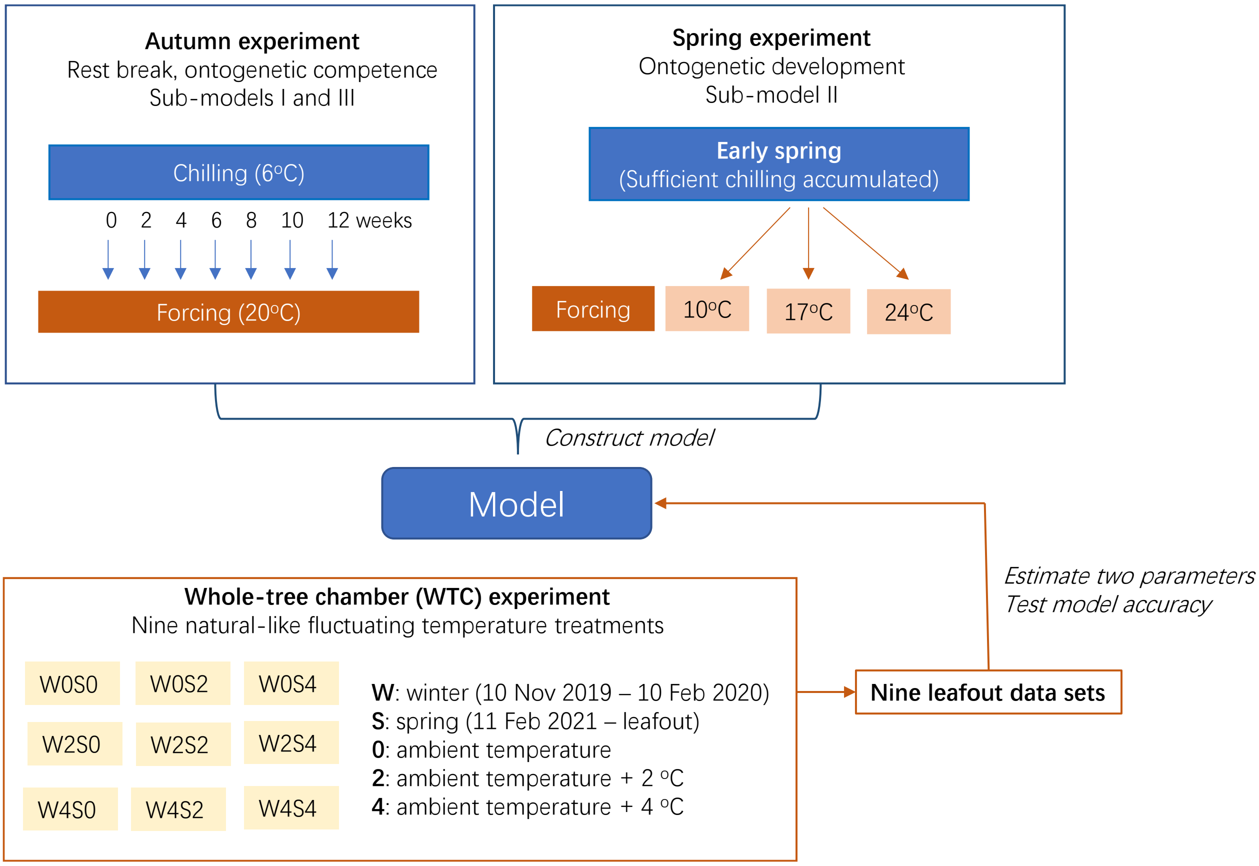

FIGURE S2. Experiments carried out in the study and their relations to the process-based leaf-out modeling. In order to estimate the timing of rest completion under natural conditions, an additional chilling treatment under natural conditions was included in the autumn experiment. This was because information on rest completion under natural conditions was needed for setting the timing of the spring experiment correctly. For the sake of clarity, the natural chilling treatment is not shown in the figure.

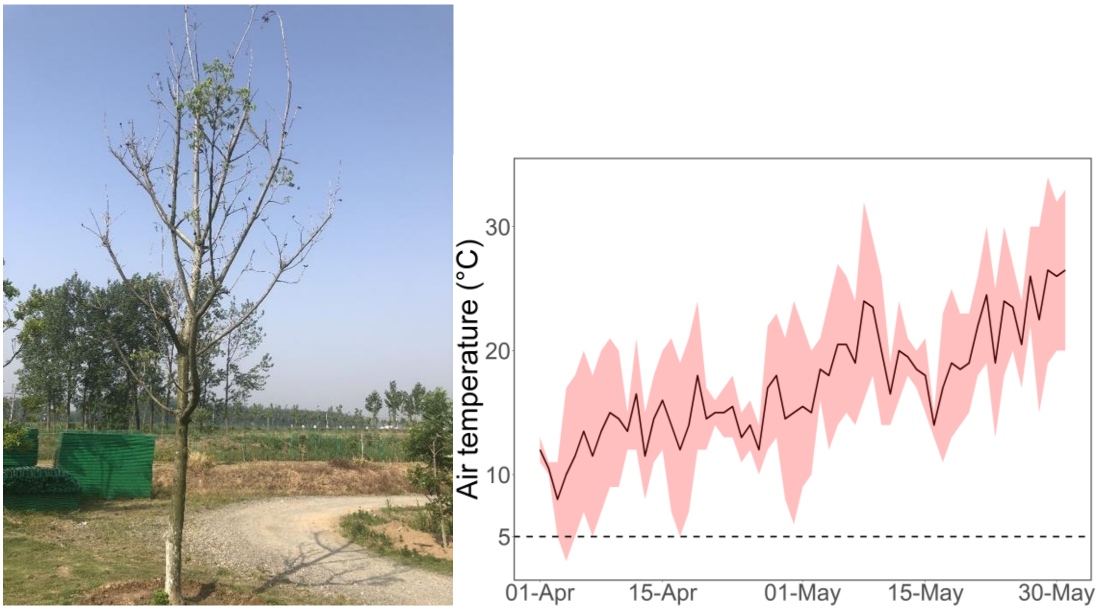

FIGURE S3. Cold damage undergone by a pecan tree growing in an orchard in Pizhou (117°35′E, 34°07′) **(A)** and the related temperature record measured in the orchard **(B)**. The curve in the middle represents the daily mean temperature and the shaded area below and above it the daily minimum and maximum temperatures, respectively. Leaf-out occurred on 17 April 2021, when the daily minimum temperature dropped to +5 ^{o}C, resulting in the cold damage seen in the photograph. On 4 April the daily minimum temperature dropped to +3 ^{o}C, but that happened more than ten days before the observed leaf-out. The photograph was taken on 30 May 2021, showing that the tree had not recovered yet.

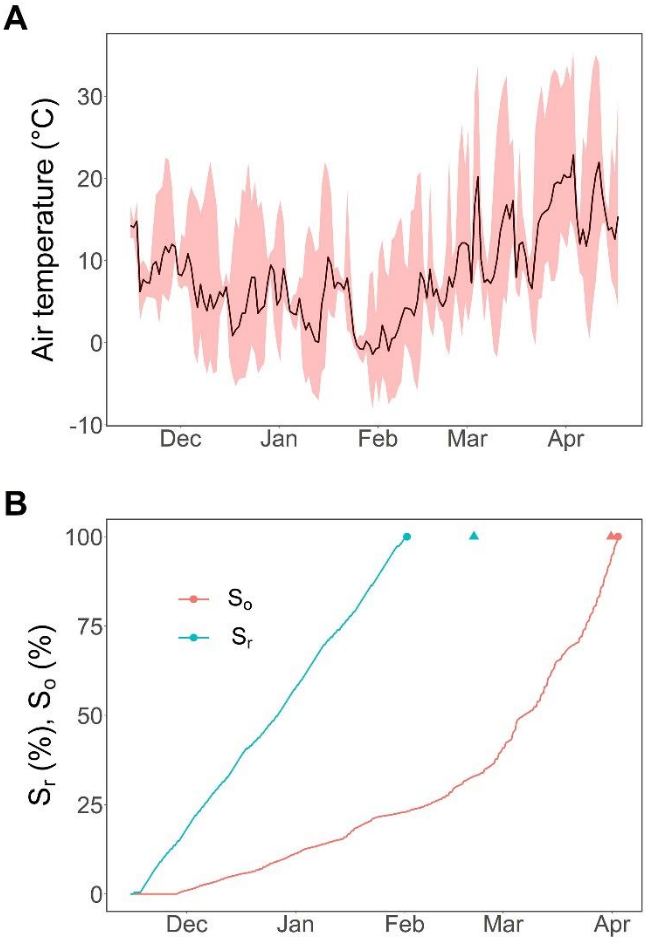

FIGURE S4. Air temperatures in natural conditions in the campus outdoor seedling collection from autumn 2017 to spring 2018 **(A)**. The curve in the middle represents the daily mean temperature and the shaded area below and above it the daily minimum and maximum temperatures, respectively. Dormancy dynamics of the first-year pecan seedlings in the campus outdoor seedling collection during the same period, as predicted by the process-based leaf-out phenology model developed in the present study **(B)**. The blue curve represents the predicted state of rest break, S_r, and the red curve the predicted state of ontogenetic development, S_o. The corresponding predicted dates of rest completion (1 February) and leaf-out (2 April) are marked with the circles at the end of the respective curve. The independent observed dates of rest completion (21 February) and leaf-out (1 April) are marked with triangles in respective colors.

TABLE S1. Sensitivity analysis of the effects of the upper threshold of the rest-breaking temperature range, T_{upp}, on the projected cold damage year percentage, CDY%, of first-year pecan (*Carya illinoinensis*) seedlings at three locations in south-eastern subtropical China under the warming scenarios RCP4.5 and RCP8.5 in 2022 – 2099. The results for the estimated value of T_{upp} (13 °C) are given in bold.

**Location**	$\mathbf{T_{upp}}$	**CDY% RCP4.5**	**CDY% RCP8.5**
Hangzhou	11	0.0	1.3
Hangzhou	12	0.0	1.3
Hangzhou	**13**	**0.0**	**3.9**
Hangzhou	14	1.3	3.9
Hangzhou	15	2.6	5.1
Hefei	11	2.6	5.1
Hefei	12	5.1	6.4
Hefei	**13**	**5.1**	**6.4**
Hefei	14	5.1	6.4
Hefei	15	6.4	7.7
Nanping	11	3.9	3.9
Nanping	12	7.7	5.1
Nanping	**13**	**16.7**	**9.0**
Nanping	14	21.8	12.9
Nanping	15	25.6	15.4

REFERENCES:

Hänninen, H. (1990). Modelling bud dormancy release in trees from cool and temperate regions. *Acta For. Fenn.* 213, 1-47. doi: 10.14214/aff.7660

Hänninen, H. (2016). *Boreal and temperate trees in a changing climate: Modelling the ecophysiology of seasonality*. Dordrect: Springer Science+Business Media. doi: 10.1007/978-94-017-7549-6

Hänninen, H., and Kramer, K. (2007). A framework for modelling the annual cycle of trees in boreal and temperate regions. *Silva Fenn.* 41, 167-205. doi: 10.14214/sf.313

Lundell, R., Hänninen, H., Saarinen, T., Åström, H., and Zhang, R. (2020). Beyond rest and quiescence (endodormancy and ecodormancy): A novel model for quantifying plant-environment interaction in bud dormancy release. *Plant Cell Environ.* 43, 40-54. doi: 10.1111/pce.13650

Zhang, Q. (2015). *Studies on the temperature change in the last 100 years over Zhejiang province* (MSci. Thesis, in Chinese). Lanzhou, Lanzhou University.

Zhang, R., Lin, J., Wang, F., Shen, S., Wang, X., Rao, Y. et al. (2021a). The chilling requirement of subtropical trees is fulfilled by high temperatures: A generalized hypothesis for tree endodormancy release and a method for testing it. *Agric. For. Meteorol.* 29:108296. doi: 10.1016/j.agrformet.2020.108296

Zhang R., Lin J., Wang F., Hänninen H., and Wu J. (2021b). Effects of climatic warming on spring phenology in subtropical trees: process-based modelling with experiments designed for model development. *Authorea*. 31:2021. doi: 10.22541/au.161210609.90934754/v1

Zhang, R., Peng, F., and Li, Y. (2015). Pecan production in China. *Sci. Horticult.* 197, 719-727. doi: 10.1016/j.scienta.2015.10.035
